# WISP1/CCN4 inhibits adipocyte differentiation through repression of PPARγ activity

**DOI:** 10.1038/s41598-017-01866-2

**Published:** 2017-05-11

**Authors:** Nathalie Ferrand, Véronique Béreziat, Marthe Moldes, Maurice Zaoui, Annette K. Larsen, Michèle Sabbah

**Affiliations:** 1 Sorbonne Universités, Cancer Biology and Therapeutics, UPMC Univ Paris 06, INSERM, CNRS, Institut Universitaire de Cancérologie, Saint-Antoine Research Center (CRSA), F-75012 Paris, France; 2 Sorbonne Universités, Genetic and Acquired Lipodystrophies, UPMC Univ Paris 06, INSERM, Hospitalo-Universitary Institute, ICAN, Saint-Antoine Research Center (CRSA), F-75012 Paris, France

## Abstract

WISP1 (Wnt1-inducible signaling pathway protein-1, also known as CCN4) is a member of the CCN family able to mediate cell growth, transformation and survival in a tissue-specific manner. Here, we report that WISP1 expression was highly increased in preadipocytes and decreased during adipocyte differentiation. Moreover, we observed an increase in WISP1 gene expression in adipose tissue from both diet-induced and leptin-deficient *ob/ob* obese mice, suggesting that WISP1 could be involved in the pathophysiological onset of obesity. Interestingly, overexpression of WISP1 in 3T3-F442A cells prevented adipocyte differentiation via downregulation of peroxisome proliferator-activated receptor (PPARγ) transcriptional activity thereby attenuating the expression of adipogenic markers. Conversely, silencing of WISP1 enhanced adipocyte differentiation. We further show that the inactivation of PPARγ transcriptional activity was mediated, at least in part, by a direct physical association between WISP1 and PPARγ, followed by proteasome-dependent degradation of PPARγ. These results suggest for the first time that WISP1 interacts with PPARγ and that this interaction results in the inhibition of PPARγ activity. Taken together our results suggest that WISP1 functions as a negative regulator of adipogenesis.

## Introduction

WISP1 (Wnt1-inducible signaling pathway protein-1, also known as CCN4) is a member of the connective tissue growth factor/cystein-rich 61/nephroblastoma overexpressed (CCN) family^1^. The CCN family is composed of six members, Cyr61/CCN1, CTGF/CCN2, NOV/CCN3, WISP1/CCN4, WISP2/CCN5 and WISP3/CCN6, referred to as CCN1-6, based on the unified nomenclature^2^. Three of these proteins, WISP1-3, were initially identified in C57MG cells, a mouse mammary epithelial cell line with Wnt-1 expression, and subsequently shown to be Wnt-1-induced genes^3^. WISP1/CCN4 (hereafter referred to as WISP1) is both an intracellular and a secreted protein found in the extracellular matrix (ECM) and, like many other matricellular proteins able to modulate cellular responses such as cell growth, differentiation and survival^4^. WISP1 is expressed in various normal tissues including heart, kidney, lung, pancreas, placenta, ovary, small intestine and spleen. Interestingly, elevated WISP1 expression has also been observed in a variety of cancers including hepatocellular carcinoma^5^, colon adenocarcinoma^3^, lung carcinoma^6^, breast cancer^7^ and cholangiocarcinoma^8^.

Wnt signaling pathway plays a key role for maintaining the cells in an undifferentiated state^9^. The Wnt proteins are secreted signaling factors that affect cell fate and differentiation, including adipogenesis, myogenesis and mammary development. When Wnt signaling is active, GSK-3β is inhibited, allowing β-catenin to accumulate in the nucleus where it binds to TCF-LEF transcription factors and activates Wnt-target genes. The Wnt-1 protein has been described as an inhibitor of adipocyte differentiation^9^. Interestingly, ectopic expression of Wnt-1 in 3T3-L1 murine preadipocytes induced the expression of several downstream genes including WISP1^10^, thereby suggesting a potential role for WISP1 in adipocyte differentiation.

Adipogenesis is characterized by dynamic changes in gene expression^11–13^. It is well accepted that both peroxisome proliferator-activated receptor γ (PPARγ) and CCAAT/enhancer binding protein (C/EBPs) function as critical regulators of adipogenesis^14, 15^. PPARs are members of the Nuclear Receptor family, comprising a subgroup of three homologous genes. Among them, PPARγ acts primarily as a master regulator of adipocyte differentiation and metabolism^15, 16^. In particular, PPARγ is sufficient and necessary for fat cell differentiation. Following ligand-mediated activation, the PPARγ receptor binds to the PPARγ response element (PPRE) thereby promoting the expression of target genes^17^. Forced expression of PPARγ can convert fibroblasts into adipocytes, whereas dominant-negative PPARγ mutants in cultured preadipocytes inhibit adipogenesis. PPARγ is the pharmacologic target of thiazolidinedione (TZD) drugs, which act as potent insulin sensitizers (for review see^18^).

In the present work, we hypothesized that WISP1 could be involved in the regulation of adipogenesis and investigated the influence of WISP1 on adipocyte differentiation. We show that both WISP1 overexpression and knock-down affects adipocyte differentiation. Our results indicate that WISP1 interacts with PPARγ, mediates proteasome-dependent degradation of PPARγ, thereby reducing PPARγ protein level and activity. Moreover, using both genetic and diet-related mouse models of obesity we found that WISP1 expression increases with obesity and may contribute to the dysregulation of adipocyte function.

## Results

### WISP1 expression decreases during adipogenesis

**Figure 1:**
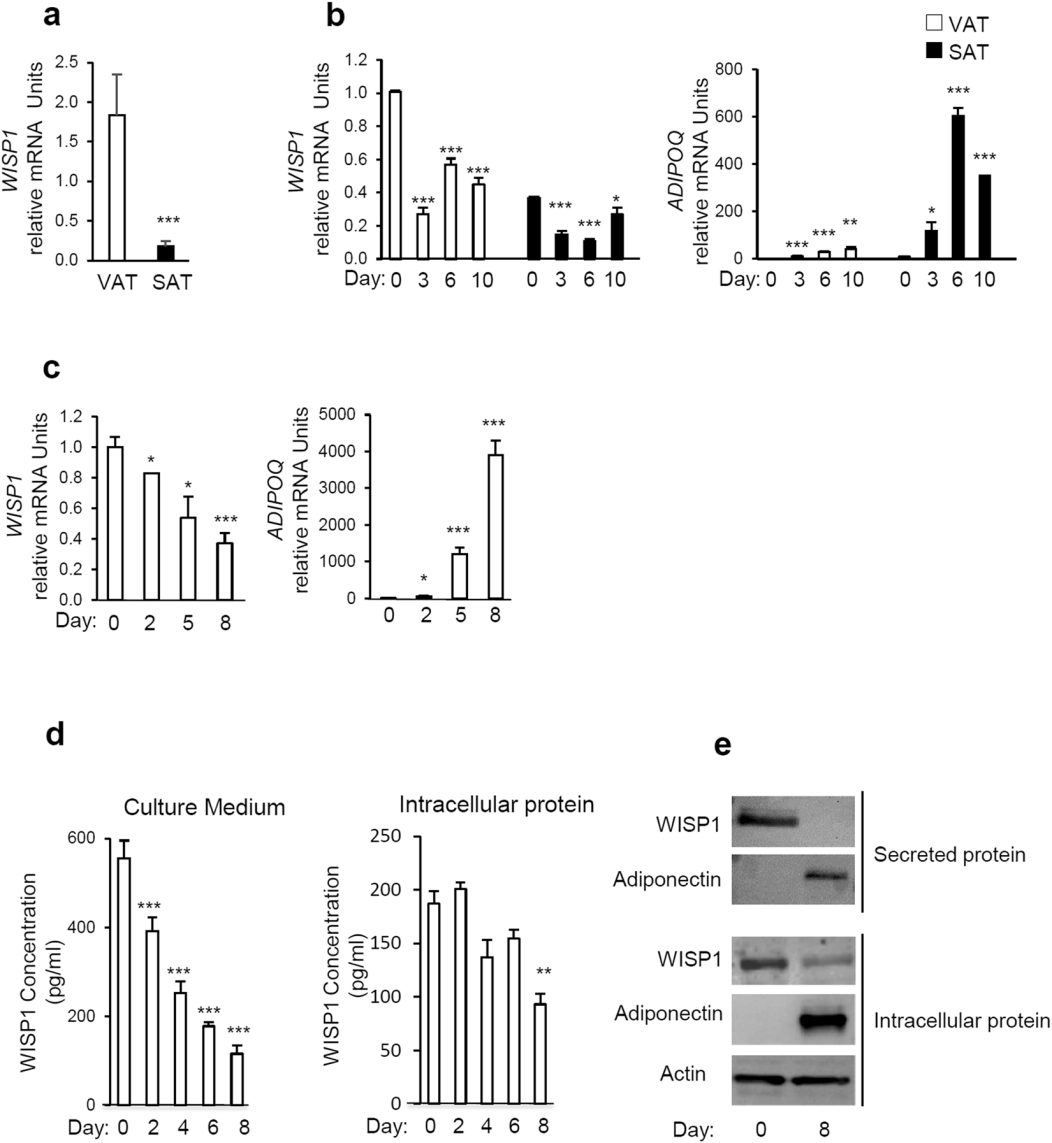
WISP1 gene expression is decreased during adipocyte differentiation. (**a**) The expression of *WISP1* mRNA was measured in subcutaneous (SAT) and epididymal adipose tissues (VAT) harvested from 12 weeks old C57BL6 mice (n = 4–5 per group). (**b**) Preadipocytes isolated from VAT and SAT were induced to differentiate. Total RNA was isolated and the expression of *WISP1* and *ADIPOQ* mRNA were measured by real-time PCR. The values indicate the changes for the indicated samples compared to VAT on Day 0. (**c**) 3T3-F442A cells were induced to differentiate and the gene expression of *WISP1*, and *ADIPOQ* was measured by real-time PCR. The values indicate the changes for the indicated samples compared to day 0. (**d**) Secreted and intracellular protein levels of WISP1 were analyzed during adipocyte differentiation by ELISA. (**e**) Expression of secreted and intracellular protein levels of WISP1 and adiponectin were measured by Western Blotting. Adiponectin serves as a positive control for adipocyte differentiation while actin was used as a loading control. All values are representative of data from 3 independent experiments each performed in duplicate. Results are presented as means ± SEM *p < 0.05; **p < 0.01; ***p < 0.005.

We first determined whether WISP1 was expressed in both visceral (VAT) and subcutaneous adipose tissues (SAT) in mice. Interestingly, our results show that the expression of *WISP1* gene was higher in VAT compared to SAT (Fig. 1a). To evaluate the expression of *WISP1* during adipogenesis, murine adipose precursor cells from the SAT or VAT adipose tissues were isolated and induced to differentiate *in vitro*. In agreement with the findings in Fig. 1a, SAT contained about three times less *WISP1* mRNA compared to VAT at confluence (Day 0, Fig. 1b). In both cases, the levels of *WISP1* mRNA decreased during adipocyte differentiation, in contrast to the expression of the *ADIPOQ* gene that increased during the same period (Fig. 1b).

Next, the expression of *WISP1* was evaluated during 3T3-F442A differentiation. As shown in Fig. 1c–e, both WISP1 mRNA content and protein secretion level, decreased significantly during differentiation in a time-dependent manner whereas the expression of adiponectin transcripts and secretion increased during the same period (Fig. 1c and e). Interestingly, we observed a slower and modest decrease of intracellular WISP1 protein (Fig. 1d and e).

### Stimulation of Wnt-signaling, but not TNF-α, increases WISP1 expression with a concomitant inhibition of 3T3-F442A adipocyte differentiation

**Figure 2:**
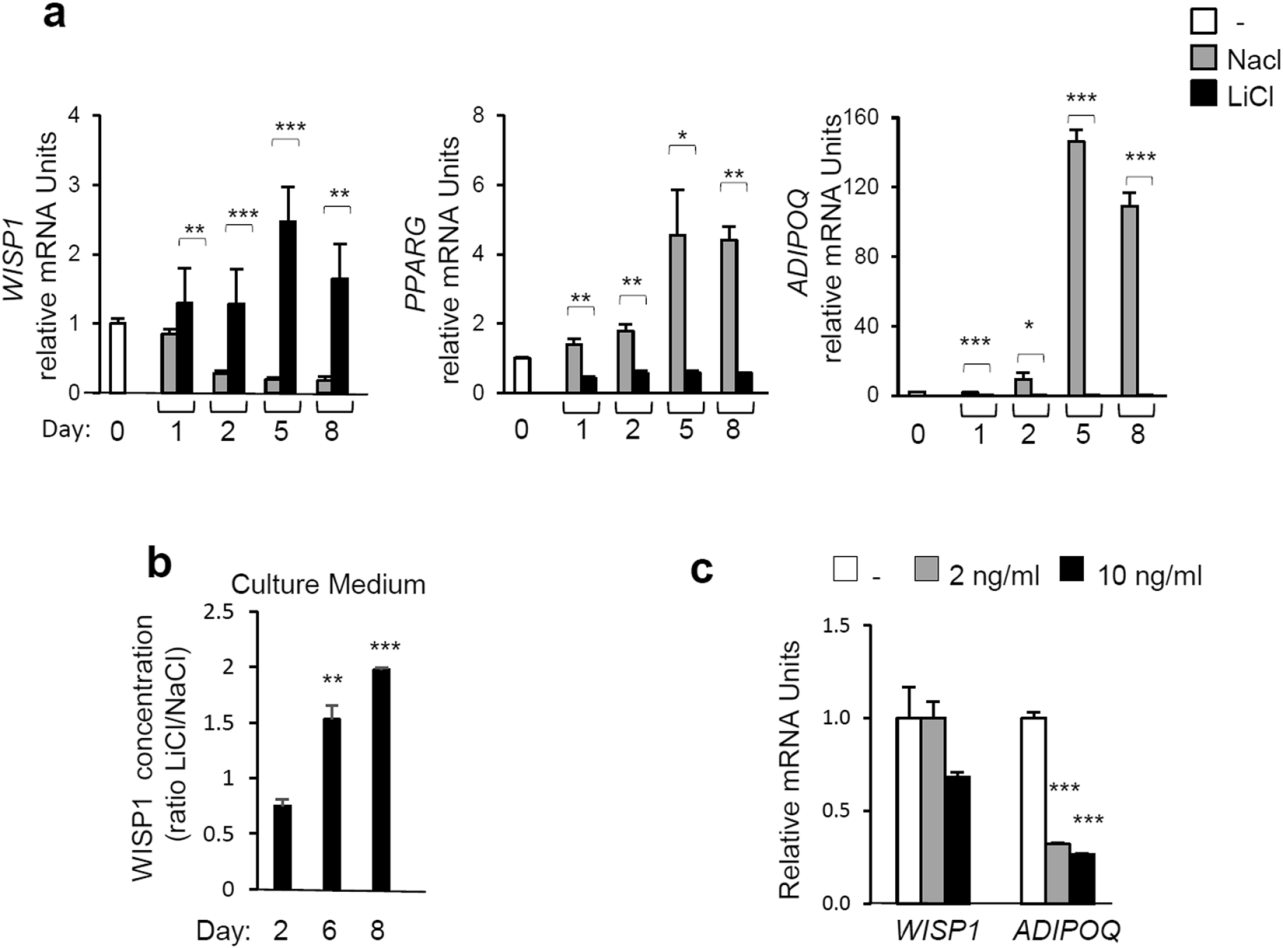
Stimulation of Wnt-signaling increases the expression of WISP1 and inhibits adipocyte differentiation. 3T3-F442A preadipocytes were exposed to MDI media supplemented with 25 mM Lithium Chloride (LiCl) or, as negative control, 25 mM Sodium Chloride (NaCl). (**a**) Gene expression of *WISP1, PPARG*, and *ADIPOQ* were measured by real-time PCR. The values indicate the changes in the indicated sample compared to day 0. (**b**) The secretion of WISP1 following activation of Wnt signaling was determined by ELISA. The concentration of WISP1 is indicated as the ratio of LiCl-treated compared to the corresponding NaCl-treated sample. (**c**) 3T3-F442A preadipocytes at two days post confluence were treated with or without TNF-α at the indicated concentrations. After 24 hours, total RNA was isolated and the expression of *WISP1* and *ADIPOQ* was analyzed by real-time PCR. The values indicate the changes for the indicated sample compared to the vehicle. All values are representative of data from 3 independent experiments each performed in duplicate. Results are presented as means ± SEM *p < 0.05; **p < 0.01; ***p < 0.005.

Negative regulators of adipogenesis include canonical Wnt-signaling^9^ and inflammatory cytokines such as tumor necrosis factor-α (TNF-α)^19^. Therefore, we examined the role of WISP1 as a mediator of canonical Wnt and TNF-α signaling. Its inhibitory effect on adipogenesis was investigated using Lithium Chloride (LiCl), a well-known activator of Wnt-signaling that acts by inhibiting the catalytic activity of GSK-3β, thereby stabilizing free cytosolic β-catenin^20^. The results show that lithium treatment prevented the differentiation of 3T3-F442A preadipocytes as shown by a drastic decrease in the expression of the *PPARG* and *ADIPOQ* genes as well as by a decrease in lipid accumulation as visualized by Oil Red O-staining (Fig. 2a, Fig. S1a). In the presence of LiCl, *WISP1* gene expression (Fig. 2a) and protein secretion levels (Fig. 2b) increased and are correlated with stabilization of active β-catenin compared to the NaCl control samples (Fig. S1b). In contrast, TNF-α did not affect the expression of *WISP1* whereas the *ADIPOQ* expression was markedly inhibited (Fig. 2c). These results suggest that WISP1 could be required for inhibition of adipogenesis by Wnt but not by TNF-α.

### WISP1 overexpression inhibits 3T3-F442A preadipocyte differentiation

**Figure 3:**
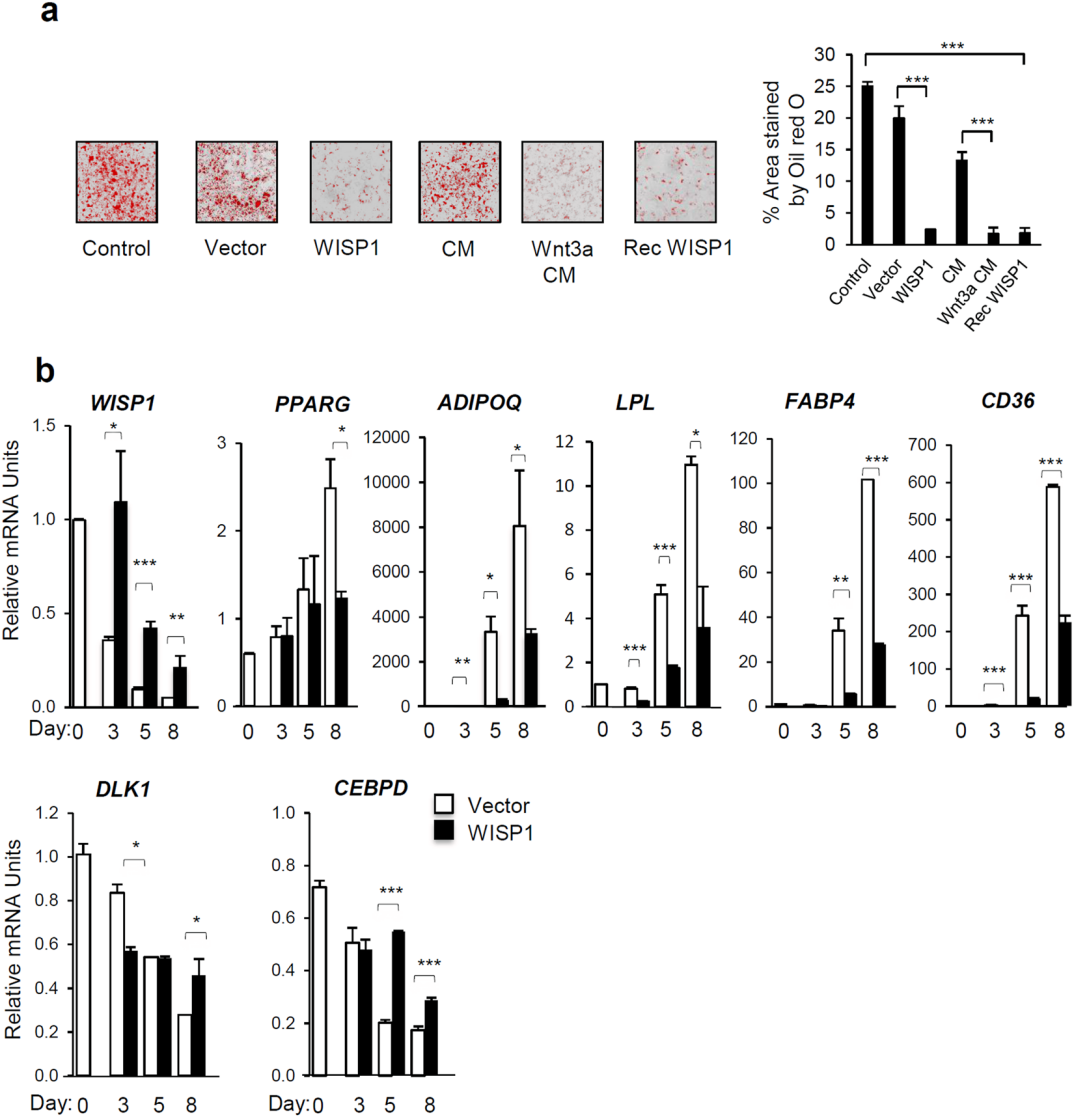
WISP1 inhibits adipogenesis. (**a**) 3T3-F442A preadipocytes were transfected or not with 2.5 µg of empty (vector) or WISP1-encoding vector (WISP1) in the presence of MDI media for 72 hours. Alternatively, cells were incubated for 72 hours in MDI media (control) in the presence of recombinant WISP1 (Rec WISP1) or conditioned media from L cells expressing (Wnt3a-CM) or not (CM) Wnt3a. The differentiation process was monitored by microscopic imaging after Oil Red O-staining. Subsequently, Oil Red O-stained areas were quantified by ImageJ analysis (right). (**b**) 3T3-F442A preadipocytes were transfected with 2.5 µg of empty vector or human WISP1-encoding vector. Three, 5 and 8 days after transfection, endogenous murine *WISP1, PPARG, ADIPOQ, LPL, FABP4, CD36, DLK1 and CEBPD* mRNA levels were quantified by real-time PCR. The values indicate the changes for the indicated sample compared to day 0. All the values are averages of data from 3 independent experiments performed in duplicate. Results are presented as means ± SEM *p < 0.05; **p < 0.01; ***p < 0.005.

To further assess the role of WISP1 during adipogenesis, we either overexpressed WISP1 or added recombinant murine WISP1 protein to undifferentiated 3T3-F442A preadipocytes. Like Wnt3a, a well-known inhibitor of adipogenesis^21^, ectopic expression of human WISP1, which is 85% homologous to the murine WISP1, or the addition of murine recombinant WISP1 during the differentiation process markedly reduced the differentiation as shown by Oil Red O-staining (Fig. 3a). Furthermore, the mRNA levels of *ADIPOQ, LPL, FABP4* and *CD36*, which are all PPARγ target genes, were reduced in the presence of WISP1 (Fig. 3b). On the other hand, mRNA content of *DLK1* and *CEBPD* which are expressed early during adipogenesis, with a maximal induction observed between 24 and 48 hr of differentiation (data not shown), was maintained throughout the course of differentiation (Fig. 3b). At day 5, the decrease in *PPARG* gene expression was marginal compared with the large decrease in PPARγ target genes (Fig. 3b). These results suggest that WISP1 functions as a negative regulator of adipocyte differentiation which is associated with inhibition of PPARγ target genes.

### WISP1 knockdown induces 3T3-F442A preadipocyte differentiation

**Figure 4:**
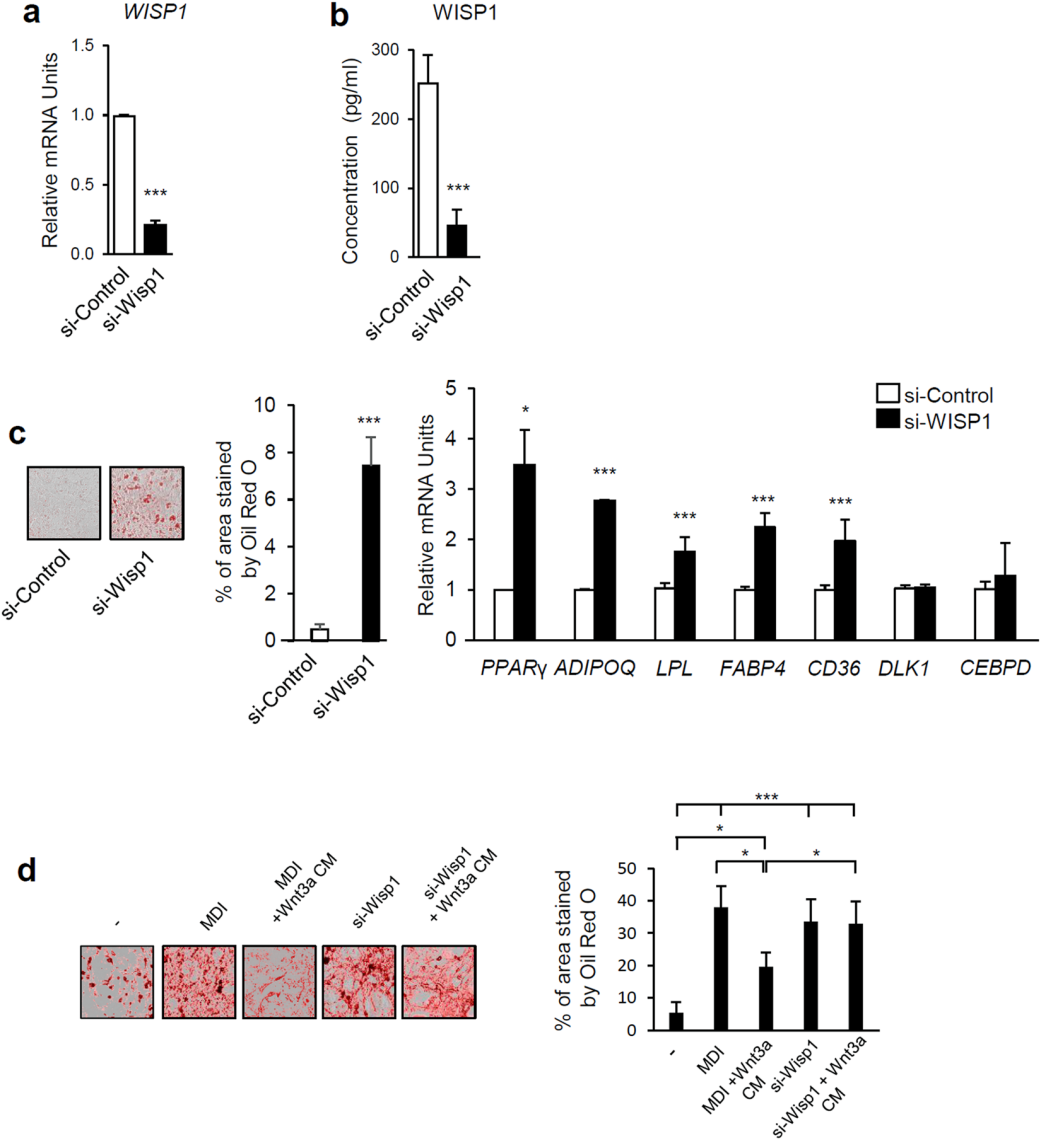
WISP1 silencing induces adipogenic differentiation. 3T3-F442A preadipocytes were transfected for 48 hours with siRNA control or siWISP1 (30 pmoles). Then, the influence of WISP1 knockdown on (**a**) endogenous *WISP1* mRNA levels and (**b**) secreted WISP1 protein levels in the culture media was determined by real-time PCR and ELISA. (**c**) The effect of WISP1 knockdown on lipid accumulation was monitored by microscopic imaging after Oil Red O-staining. mRNA levels of *PPARG, ADIPOQ, LPL, FABP4, CD36, DLK1 and CEBPD* were measured by real-time PCR. The values indicate the changes for the indicated sample compared to the siRNA-control. (**d**) 3T3-F442A preadipocytes were incubated in MDI media or alternatively transfected with si-WISP1 (30 pmoles) in the presence or the absence of conditioned media from L cells expressing Wnt3a (Wnt3a-CM) for 96 hours. The differentiation process was monitored by microscopic imaging after Oil Red O-staining. Red-stained areas were quantified by ImageJ analysis. All values are averages of data from 3 independent experiments each performed in duplicate. Results are presented as means ± SEM; *p < 0.05; **p < 0.01; ***p < 0.005.

To further validate the influence of WISP1 on adipocyte differentiation, we transfected 3T3-F442A preadipocytes with si-RNA targeting *WISP1*. Knockdown of endogenous WISP1 resulted in 80% decrease for both mRNA and secreted protein (Fig. 4a,b). Importantly, the attenuation of WISP1 induced increased formation of lipid droplets, concomitant with a significant increase in the expression of PPARγ (~3–4-fold) and its target genes expression *ADIPOQ, LPL*, *FABP4* and *CD36 (*~2–3-fold) (Fig. 4c). No difference was observed on the expression of *DLK1* and *CEBPD* during early adipogenesis (Fig. 4c). To further validate the role of WISP1 as a mediator required for the wnt-associated inhibition of adipogenesis, we added Wnt3a together with si-RNA targeting WISP1 in 3T3F442A cells. Whereas Wnt3a prevented differentiation of control cells, cells lacking WISP1 underwent adipogenesis despite the presence of Wnt3a (Fig. 4d). These results suggest that WISP1 plays a role in Wnt inhibitory effect on adipogenesis.

### WISP1 decreases PPARγ transcriptional activity

**Figure 5:**
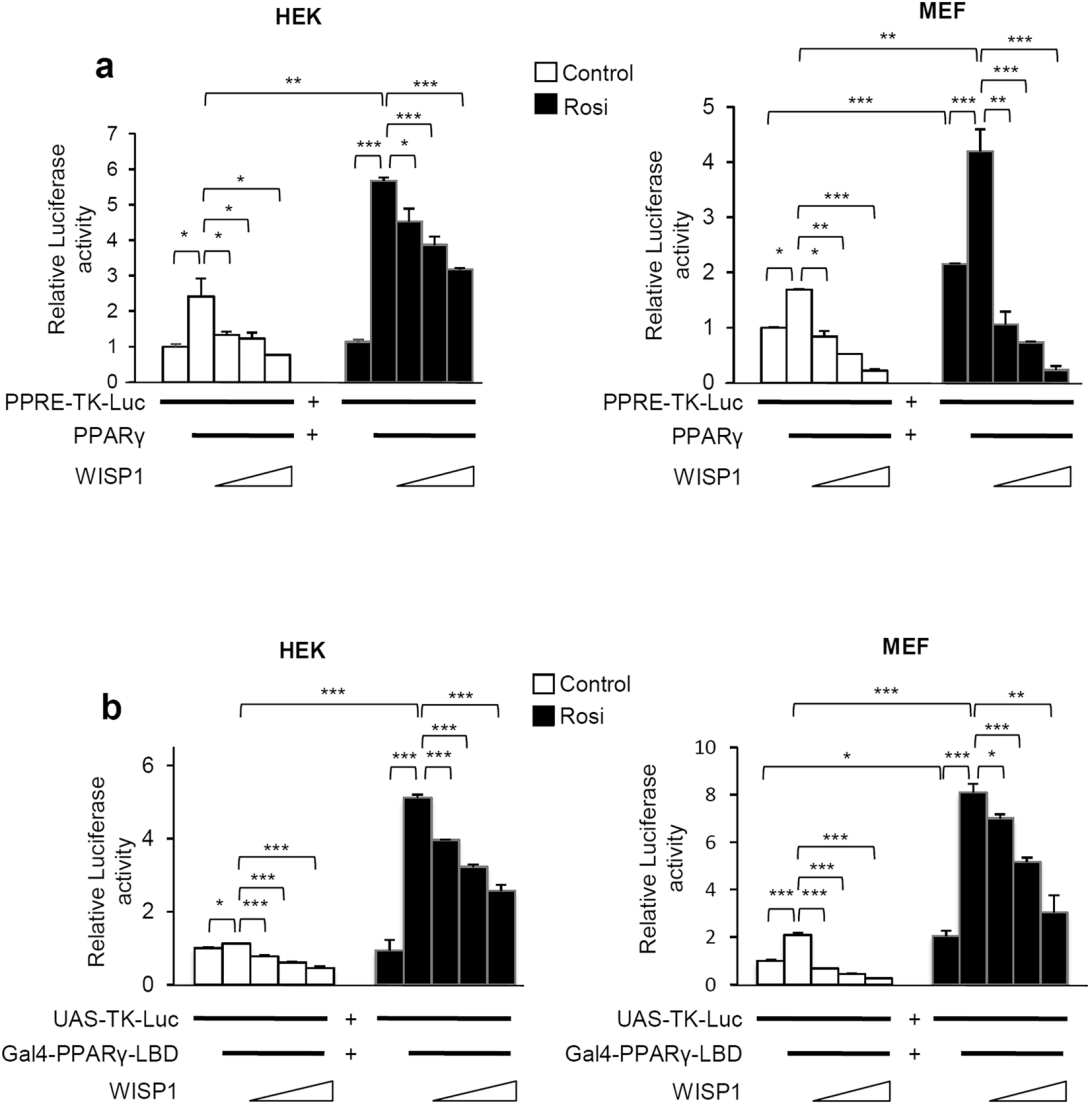
WISP1 decreases PPARγ transcriptional activity. (**a**) Increasing amounts of expression vector for WISP1 (0.2, 0.4, 0.8 µg) were transfected into HEK or MEF cells along with 0.1 µg of PPRE-TK-Luc and 0.02 µg of an RSV-β-galactosidase construct as an internal control, alone or in combinations with 0.1 µg of PPARγ expressing vector. (**b**) Increasing amounts of WISP1 (0.2, 0.4, 0.8 µg) were transfected into HEK and MEF cells along with 0.1 µg of UAS-TK-Luc and 0.02 µg of an RSV-β-galactosidase construct as an internal control, alone or in combination with 0.1 µg of an expression vector for the Gal4-PPARγ-LBD fusion protein. Five hours after transfection, cells were treated (black bars) or not (open bars) with 1 µM rosiglitazone for 24 hours and assayed for luciferase and β-galactosidase activities. The results represent the average of at least three independent experiments each done in triplicate. Results are presented as means ± SEM; *p < 0.05; **p < 0.01; ***p < 0.005.

To elucidate the molecular mechanisms underlying the influence of WISP1 on adipogenesis, we determined if there was a functional connection between WISP1 and PPARγ, a master regulator of adipocyte differentiation. Co-transfection experiments using a PPARγ-responsive luciferase reporter construct (PPRE-TK-Luc) and expression vectors for PPARγ and WISP1 in HEK and MEF cells were performed. PPARγ induced the luciferase activity by 2.4- and 1.7-fold in both HEK and MEF cells, which is further enhanced by almost 6- and 4.2-fold in the presence of PPARγ agonist rosiglitazone, respectively (Fig. 5a). As expected, increasing amounts of WISP1 significantly prevented PPRE- luciferase activity even in the presence of rosiglitazone (Fig. 5a). Mutation of the PPRE site in the PPRE-TK-Luc construct (mPPRE-TK-Luc) totally prevented the WISP1 inhibition showing the specificity of PPARγ effect on its PPRE site (Fig. S2). To further determine the direct effect of WISP1 on PPARγ transcriptional activity, we used a Gal4-responsive luciferase reporter (UAS-TK-Luc) and a plasmid expressing the Gal4-PPARγ-LBD fusion protein, which contains the Gal4 DNA binding domain fused to the ligand-binding domain (LBD) of PPARγ. As shown in Fig. 5b, the Gal4-PPARγ-LBD-fusion protein is able to induce the UAS-TK-Luc in the presence of rosiglitazone (≥5-fold and 8-fold induction, respectively, in HEK and MEF cells). In comparison, WISP1 was able to decrease both basal and ligand-induced PPARγ activity in a dose-dependent manner (Fig. 5b), clearly demonstrating that WISP1 acts as a negative regulator of PPARγ transcriptional activity.

### WISP1 interacts with PPARγ and induces its proteosomal degradation

**Figure 6:**
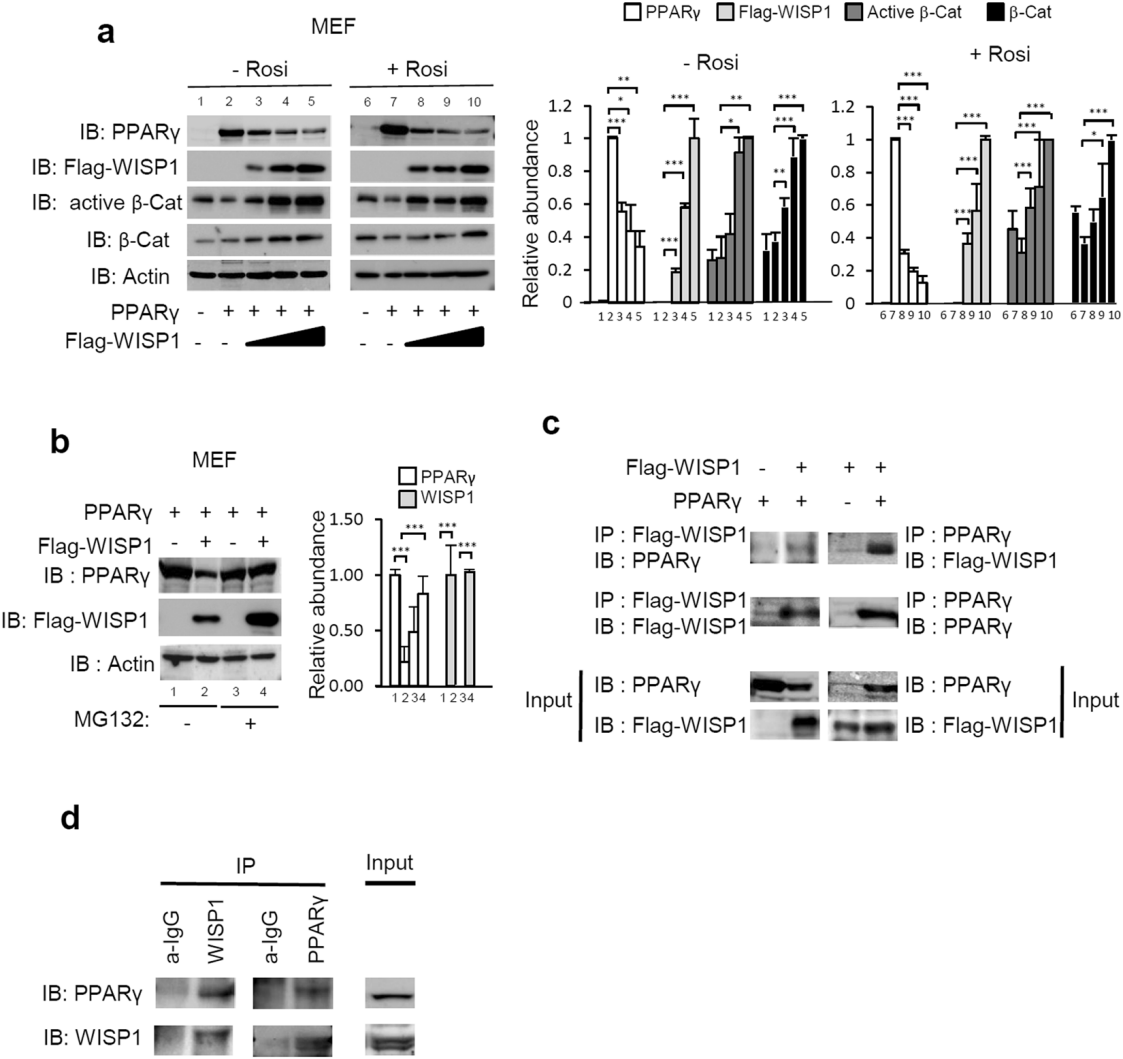
WISP1 interacts with and down-regulates PPARγ (**a**) MEF cells were transfected with increasing amounts of Flag-WISP1 expression vector (0.65, 1.30 and 2.60 µg) in combination with 1 µg of PPARγ expression vector. After 4 hours, cells were treated with 1 µM of rosiglitazone and harvested 24 hours later. Lysates were subjected to immunoblotting with anti-PPARγ, anti-Flag, anti-active β-catenin or anti-β-catenin antibodies. Immunoblot signals were quantified by densitometry, and normalized with β-actin. Data were expressed as means ± SEM; *p < 0.05; **p < 0.01; ***p < 0.005. Data are representative of 3 independent experiments (**b**) MEF cells were transfected with 2 µg of Flag-WISP1 expression vector in combination with 2 µg of PPARγ expression vector. After 24 hours, cells were treated with 10 µM MG132 for 6 hours and harvested. Lysates were subjected to immunoblotting with anti-PPARγ and anti-Flag antibodies. Immunoblot signals were quantified by densitometry, and normalized with β-actin. Data were expressed as means ± SEM; *p < 0.05; **p < 0.01; ***p < 0.005. Data are representative of 3 independent experiments (**c**) MEF cells were transfected with 2 µg of Flag-WISP1expression vector in combination with 2 µg of PPARγ expression vector for 24 hours. Lysates were subjected to anti-Flag or anti-PPARγ immunoprecipitation (IP) and followed by immunoblotting (IB) with anti-PPARγ or anti-Flag antibodies respectively. Protein expressions were determined by direct immunoblotting (Input). Two independent experiments were performed. (**d**) MEF cells lysates were subjected to anti-WISP1 or anti-PPARγ immunoprecipitation (IP) and followed by immunoblotting (IB) with anti-PPARγ or anti-WISP1 antibodies, respectively. Immunoprecipitations with IgG were used as controls. Protein expressions were determined by direct immunoblotting (Input). Two independent experiments were performed.

To determine how WISP1 modulates PPARγ activity, MEF cells were co-transfected with increasing concentrations of Flag-tagged WISP1 and PPARγ expression vectors in the presence or absence of rosiglitazone. After 48 hours, cells were harvested and cell lysates were analyzed for the WISP1 and PPARγ protein content. The results show that WISP1 decreased the expression of PPARγ in a dose-dependent manner both in the presence or absence of rosiglitazone (Fig. 6a). To determine whether the WISP1-associated down-regulation of PPARγ involves the proteasome system, the same experiment was performed in the presence or absence of the selective proteasome inhibitor MG132. The results show that in the presence of MG132, WISP1 can no longer induce the down-regulation of PPARγ protein content (Fig. 6b). To get further, ubiquitination analyses were carried out and showed an increase in PPAR*γ* ubiquitination in the presence of WISP1 under treatment with MG132 (Fig. S3).

Because PPARγ is known to interact with β-catenin and to induce its proteosomal degradation^22^, we explored the possibility that WISP1 could induced the dissociation of the PPARγ-β-catenin complex leading to increased levels of active β-catenin. As shown in Fig. 6a, increasing amounts of Flag-WISP1 leads to a significant increase of the active form of β-catenin (Fig. 6a). To address whether WISP1 is able to activate the Wnt signaling directly, recombinant WISP1 was added to a TOP/FOP reporter system. However, the addition of WISP1 was not accompanied by any detectable activation (data not shown).

To determine if WISP1 directly interacts with PPARγ, co-immunoprecipitation experiments were carried out following transfection of Flag-tagged WISP1 and PPARγ expression vectors in MEF cells. Western blot analysis of the immunoprecipitates, using anti-Flag and anti-PPARγ antibodies, strongly suggest that the two proteins are able to interact directly (Fig. 6c). This interaction was further confirmed by co-immunoprecipitation of the endogenous WISP1 and PPARγ protein (Fig. 6d). Since WISP1 is both a cytosolic and a secreted protein, we examined the localization of WISP1 and PPARγ in transfected MEF cells. As seen in Fig. S4, immunofluorescence analysis show colocalization of PPARγ and WISP1 in the cytoplasm.

### WISP1 expression is up-regulated in adipose tissues from obese mice

**Figure 7:**
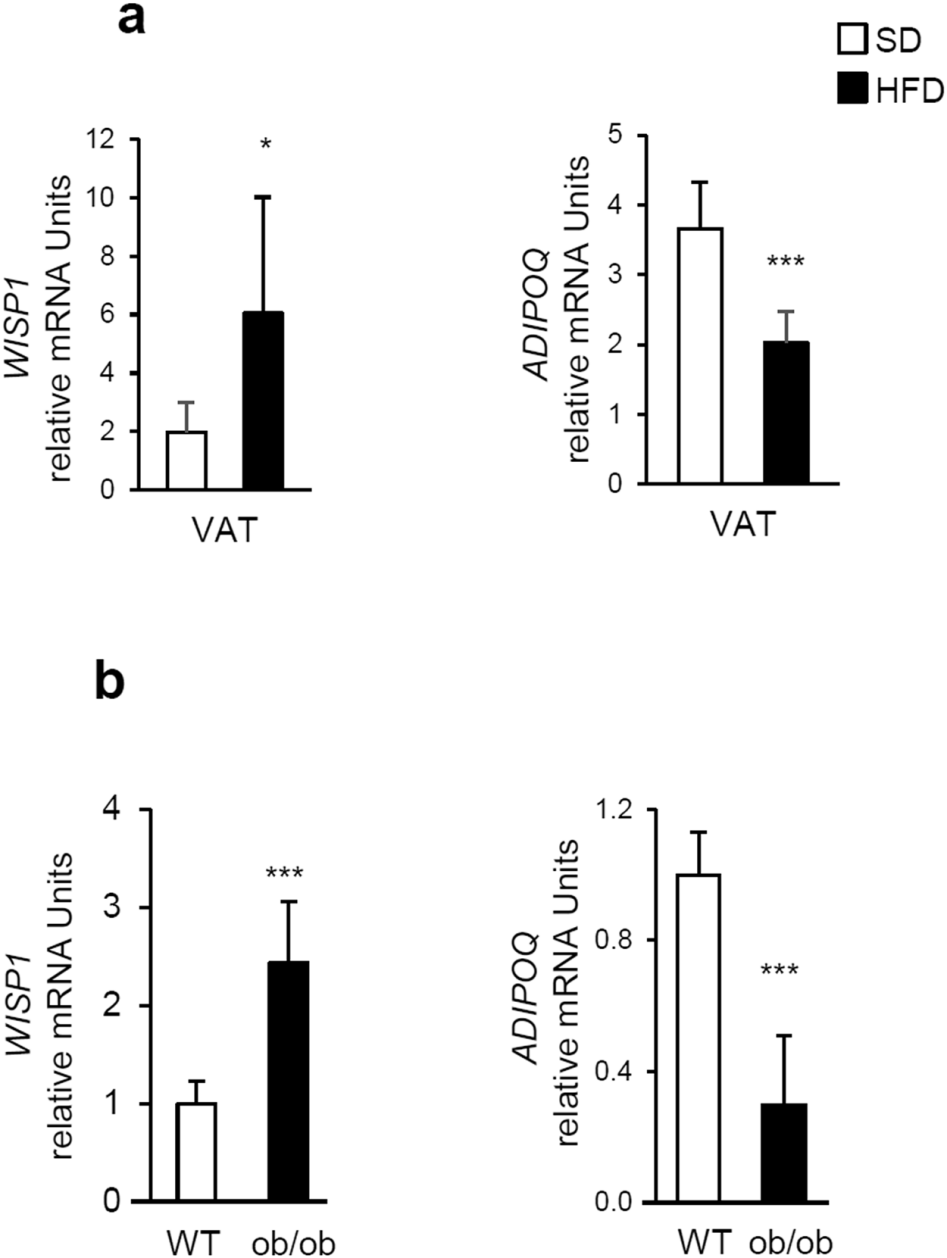
WISP1 expression is up-regulated in adipose tissues from obese mice.(**a**) *WISP1* and A*DIPOQ* mRNA levels were quantified by RT-qPCR in visceral (epididymal) adipose tissue (VAT) from mice fed with a standard diet (SD) and a high fat diet (HFD) for 12 weeks (n = 7 animals per group). (**b**) *WISP1* and *ADIPOQ* mRNA levels were quantified in VAT from ob/ob mice (n = 7 animals per group). Results are presented as means ± SEM *p < 0.05; ***p < 0.005 versus SD fed mice in panel (a) and versus WT mice in panel (b).

During obesity, adipose tissues display decreased expression of adipogenic genes^23^. Since WISP1 knockdown was associated with increased expression of adipogenic genes, we speculated that *WISP1* gene expression in adipose tissues might increase during obesity. To test this hypothesis, we evaluated the expression of *WISP1* in the adipose tissues of both diet-induced and genetic *ob/ob* murine models of obesity. The results show that transcriptional levels of *WISP1* in the VAT was increased in C57Bl/6 mice fed with a high-fat diet for 12 weeks as compared to mice fed with normal chow diet, in contrast to *ADIPOQ* gene expression. (Fig. 7a). Similar results were obtained for leptin-deficient *ob/ob* mice (Fig. 7b).

## Discussion

We here describe a novel biological function for WISP1 as a regulator of adipogenesis. Specifically, WISP1 overexpression inhibited adipocyte differentiation whereas loss of WISP1 promoted it. We further show for the first time that WISP1 interacts physically with PPARγ and attenuates the transcriptional activity of PPARγ in a proteasome-dependent mechanism.

As previously mentioned, WISP1 is a member of the CCN protein family. Like other members of the CCN family, WISP1 has four defined structural motifs that are conserved in all family members except for WISP2/CCN5 that lacks the last structural domain. The four domains are 1) Insulin-like growth factor-binding protein, 2) von Willebrand type C repeat, 3) thrombospondin type 1 repeat, and 4) cysteine-rich terminus. Considering the structural homology between the CCN members, it is not surprising that they share overlapping functions, including roles in cell migration, proliferation and regulation of signal transduction^24^. WISP1 is mainly expressed during organ development and under pathological conditions, such as fibrosis or cancer^25^. However, the exact functions of WISP1 are not well understood and current evidence suggest that WISP1 acts in a highly cell-type specific manner. Besides WISP1, other CCN family members have been linked to adipogenesis and obesity. In particular, CTGF and WISP2 have been shown to inhibit adipocyte differentiation^26, 27^. Both NOV and WISP2 are highly correlated with obesity^28, 29^. In the present study, we show that WISP1 is expressed in adipose tissue. Interestingly, although both WISP1 and WISP2 factors are established Wnt1-regulated genes, their relative expression is different in VAT and SAT. WISP1 is more expressed in VAT in both mice and human^30^ in contrast to WISP2 which is preferentially expressed in SAT^26^. This observation indicate that the expression of WISP genes is subject to multiple levels of control.

It is interesting that the effect of WISP1 on adipogenesis in mice was opposite to what has been reported in human. Using human mesenchymal stem cell-derived adipocytes, Murahovschi *et al*. found that mRNA and protein WISP1 expression increased during adipocyte differentiation^30^. In the current study, we shown that the expression of WISP1 during adipogenesis is dynamic with the lowest expression observed in fully differentiated cells, as observed in both murine preadipocytes isolated from SAT and VAT and in the murine 3T3-F442A preadipocyte cell line. Although initially surprising, our findings are in full agreement with previous reports concerning adipogenic regulators. For example, recent findings indicate that estrogen sulfotransferase inhibits adipocyte differentiation in mice but promotes adipogenesis in humans^31, 32^. In contrast, Mesoderm-specific transcript (MEST) acts as an inhibitor of human adipogenesis but stimulates murine adipogenesis^33^. Finally, the lim domain only 3 (LMO3) gene seems to be specifically induced during human adipogenesis^34^. These findings support the presence of species-specific regulatory circuits, which shares common regulatory elements with the potential to move the differentiation process in either direction. The mechanism for this species-specific effect of WISP1 on adipogenesis remains to be identified. Our observations underscore the importance of using human and mouse cells in understanding adipogenesis.

We further reveal a potential role of WISP1 in obesity consistent with its upregulation in the epididymal adipose tissue from mice on a high fat diet according to^30^ as well as in leptin-deficient *ob/ob* mice. Because this gene expression change was observed in both HFD and ob/ob mice, it was clear that the change is not the result of a specific genetic background. More likely, these data suggest that WISP1 expression in adipose tissue could play a critical role in the development of obesity. Recent data suggest that, extracellular matrix proteins are highly secreted during obesity, leading to remodeling of a fibrotic adipose tissue^35^. As a multifunctional protein, WISP1 would be able to regulate local inflammation, as well as adipose tissue remodeling, metabolism and adipogenesis during obesity.

Since PPARγ is a master regulator of adipocyte differentiation^36^, the expression of PPARγ would be tightly controlled at both the transcriptional and post-translational levels^18, 37, 38^. Transcriptional pathways that control the expression of PPARγ have been extensively studied^11^. Previous studies showed that PPARγ is polyubiquitinated and degraded in a proteasome-dependent manner^39^. We here show an additional mechanism of PPARγ regulation in which WISP1 is not only secreted but also expressed in the cytosol where it catalyzes ubiquitination and subsequent degradation of PPARγ through direct interaction, consequently inhibiting its transcriptional activity. Even in the presence of rosiglitazone, WISP1 is still able to counteract PPARγ activation and induction of adipogenesis, suggesting that WISP1 is a new potent inhibitor of adipogenesis.

Interestingly, a different functional interaction between PPARγ and Wnt-signaling pathway has already been described^40^. Indeed, PPARγ is able to suppress Wnt-signaling by targeting phosphorylated β-catenin for proteasomal degradation^22^, thereby suppressing its inhibitory action on adipogenesis. Our results suggest that besides the inhibitory impact on PPARγ adipose target genes, WISP1 could also protect β-catenin from proteasomal degradation by sequestering PPARγ, thereby promoting Wnt signaling pathway activity. Thus a bi-directional negative regulation could exist between the PPARγ and the Wnt-signaling pathways. Although WISP1 is not a direct activator of canonical Wnt signaling, as shown for WISP2^41^, WISP1 could be a novel activator of canonical Wnt signaling in adipose tissue.

In summary, our data demonstrate that WISP1 interacts with PPARγ and that this interaction results in the inhibition of PPARγ activity, thereby modulating its effect on adipocyte differentiation.

## Methods

### Animals

Three months-old male C57Bl6 mice were fed ad libitum with either a high-fat, high carbohydrate diet (HFD) (42% kcal fat and 31% kcal carbohydrates, D12451, Snniff Spezialdiäten GmbH, Soest, Germany) or a standard chow diet (SD) for 12 weeks. Ten-week-old male C57BL/6 and leptin-deficient C57BL/6 ob/ob mice were fed with a standard diet. At the end of the experiment, after 6-hours fasting, mice were anesthetized and euthanized by cervical dislocation, and adipose tissue was removed, weighted and immediately frozen in liquid nitrogen and stored at −80 °C for RNA extraction. The animal facility was granted approval (B-75-12-01) given by the French Administration. All experiments were conducted according to the European Communities Council Directive (2010/63/UE) for the care and use of animals for experimental procedures and complied with the regulations of the French Ethics Committee in Animal Experiment ≪ Charles Darwin ≫ registered at the ≪ Comité National de Réflexion Ethique sur l′Experimentation Animale ≫ (Ile-de-France, Paris, no 5). All procedures were approved by this committee (no #Ce5/2012/091). All efforts were made to minimize suffering.

### Adipose tissue fractionation

Subcutaneous adipose tissue (SAT) and visceral-epididymal tissue (VAT) were removed and cultured after isolation of the stromal vascular fraction. Briefly, the white adipose tissue (WAT) was bilaterally removed, weighed and placed in RPMI 1640, containing 2.5% BSA, and subjected to enzymatic digestion with collagenase (1 hour at 37 °C) in order to obtain a single-cell suspension. After digestion, the centrifuged cell pellet, termed the stromal vascular fraction, was filtered through 70–100 μm nylon filters. The filter was washed with RPMI 1640 media and centrifuged for 10 min at 1500 rpm. The lower phase, corresponding to stromal vascular fraction (containing adipose precursors), was centrifuged for 5 min at 1500 rpm and the cells were suspended in RPMI media containing 10% calf serum, L-Glutamine and antibiotics (non-adipogenic medium). Cells were grown to confluence and induced to differentiate into adipocytes as follows. Two days after post-confluence, the media was changed to RPMI media supplemented with 10% fetal bovine serum (FBS) containing 100 µM isobutyl methylxanthine, 100 nM dexamethasone and 170 nM insulin. After an additional 48 hours, the media was changed to RPMI media supplemented with 10% FBS containing 160 nM insulin.

### Cell culture and adipocyte differentiation

Human embryonic kidney 293 T cells (HEK) and Mouse embryonic fibroblast cells (MEF) were maintained in Dulbeco’s modified Eagle medium (DMEM), supplemented with 10% FBS, L-Glutamine, and antibiotics. Mouse preadipocyte 3T3-F442A cells were grown in DMEM containing 10% calf serum, L-Glutamine, 176.4 µM biotin, 85 µM pantothenic acid and antibiotics (non-adipogenic media). Confluent preadipocytes were differentiated into adipocytes by treatment with DMEM containing 10% FBS, L-Glutamine and antibiotics, supplemented with 170 nM insulin. This media, named media differentiation induction (MDI) was replaced every two days. All cell lines were regularly tested for mycoplasma contamination. For activation of PPARγ activity, cells were exposed to 1 µM rosiglitazone. Proteasome inhibition was achieved by treating cells for 6-hours with 10 µM MG132. Oil Red O-staining was performed as described^22^ and cells were photographed using phase-contrast microscopy. The red-stained images were quantified using ImageJ (National Institutes of Health).

### Plasmids

The entire open reading frame of WISP1 cDNA obtained from the genomic DNA of SKBR3 cells was inserted into the pCEP4-Flag vector (Invitrogen) as described^42^ and referred to as pCEP4-Flag-WISP1. Small interfering RNA (siRNA) oligonucleotides for WISP1 were a pool of 3 target-specific 19–25 nt siRNAs designed to knock-down gene expression (Santa Cruz Biotechnology). Control siRNA (Santa Cruz Biotechnology) was used as a negative control for evaluating RNAi off-target effects. Plasmids for PPRE-TK-Luc, mPPRE-TK-Luc, UAS-TK-Luc, Gal4-PPARγ-LBD and PPARγ have been described previously^43^ and were kindly provided by Dr. Lluis Fajas (University of Lausanne, Department of Physiology, Switzerland) and Dr. Nathalie Hennuyer (Institut Pasteur de Lille, France).

### Transient transfection and luciferase assays

For luciferase reporter gene assays, cells were seeded in 12-well-plates at a 1 × 10^5^ cells/well and transfected with plasmids by using Fugene HD (Promega) for HEK cells or Lipofectamine reagent (Invitrogen) for MEF cells. After 5-hours incubation, cells were treated or not with 1 µM rosiglitazone. Luciferase activity was measured (Promega assay system) 24 hours later and was normalized for transfection efficiency using a galactosidase-expressing vector and the Galacto-Star system (PerkinElmer) as described^44^. For overexpression and siRNA experiments, 3T3-F442A preadipocytes were seeded in 6-well-plates at 1.5 × 10^5^ cells/well, and transfected with plasmids using Lipofectamine Plus® reagent or with siRNAs using the Lipofectamine® RNAiMAX procedure (Invitrogen). MEF cells seeded at 5 × 10^5^ cells/well and were transfected by the Lipofectamine method.

### Western blot and ELISA

Cell extracts of MEF cells, HEK cells and of 3T3-F442A adipocyte were obtained after lysis with RIPA buffer (0.5% Sodium Deoxycholate, 50 mM Tris-HCl; pH 8, 150 mM NaCl, 1% NP40, 0.1% SDS). Adipocyte culture media collected during adipocyte differentiation and cell extracts were supplemented with protease inhibitor cocktail (Merck Millipore), and equal amounts of protein (100 μg/lane) were loaded onto SDS-PAGE gels. After transfer onto nitrocellulose membrane, blots were incubated overnight at 4 °C with anti-WISP1 antibody (1/250, Santa Cruz Biotechnology), anti-β-catenin antibody (1/1000, Santa Cruz Biotechnology), anti-actin-HRP antibody (1/2000, Santa Cruz Biotechnology), anti-adiponectin antibody (1/1000, Pierce), anti-active-β-catenin antibody (1/1000, phospho-β-Catenin (Ser675), Cell Signaling) or anti-Flag-M2 (1/1000, Sigma) and followed by incubation with a horseradish peroxidase-conjugated secondary antibody (1/2000, Cell Signaling). Bands were revealed with an enhanced chemiluminescence detection system (Millipore) and visualized on Chemidoc systems (Biorad)^45^. Protein expression was quantified by densitometric analysis of the immunoblots using Image Lab software developed by Bio-Rad. For quantification of WISP1 in the conditioned cell culture supernatant, sandwich ELISA were performed using isoform-specific capture and detection antibodies (R&D Systems) according to the manufacturers’ instructions.

### Immunoprecipitation and ubiquitination assay

Cells extracts were prepared using RIPA buffer supplemented with protease inhibitor cocktail (Merck Millipore) and 1 mM PMSF, cleared by centrifugation, and incubated with PPARγ or Flag antibodies overnight, followed by adsorption to Sepharose-coupled protein G for 1 hour. Immune complexes were washed five times with TNMG buffer (20 mM Tris; HCl pH 8, 150 mM NaCl, 5 mM MgCl_2_, 0,5% NP40, 10% Glycerol), separated by SDS-PAGE, and analyzed by immunoblotting.

In the ubiquitination assay, the P4D1 ubiquitin-directed antibody was used (Santa Cruz Biotechnology).

### Immunfluorescence

MEF cells were grown on round glass coverslips and transfected with Flag-WISP1 and PPARγ expression vectors by using Lipofectamine reagent (Invitrogen). After 48 hours, cells were fixed for 20 min at room temperature with 4% paraformaldehyde, permeabilized in 0.2% Triton-X 100 for 10 min, and blocked with 1% bovine serum albumin. Primary antibodies (mouse anti-Flag M2, Sigma; Rabbit anti-PPARγ, Santa Cruz Biotechnology) were applied at 4 °C overnight. The cells were washed and labeled with fluoresceine-conjugated anti-mouse (Jackson ImmunoResearch Laboratories) or cyanine3-conjugated anti-Rabbit (Jackson ImmunoResearch Laboratories), respectively, for 1 h. Nuclei were stained with 1 μg/ml 4′,6′-diamidino-2-phenylindole (DAPI) in PBS for 1 min at room temperature. The cells were visualized with an Olympus microscope BX61.

### Production of and treatment with Wnt3a-conditioned media

Wnt3a-conditioned media (Wnt3a-CM) and control-conditioned media (CM) were prepared as described^46^. Briefly, Wnt3a-producing L cells or control L cells were seeded at a density of 10^6^ cells in a 100 mm culture dish containing DMEM supplemented with 10% FBS and cultured then for 24 hours. Then, cells were rinsed with PBS and cultured for an additional 72 hours in serum-free DMEM. Subsequently, the culture media was centrifuged at 1000 g for 10 min and filtered through a nitrocellulose membrane. 3T3-F442A cells were treated with a mix of 50% Wnt3a- or control conditioned media supplemented with 50% MDI media.

### Real-time RT (reverse transcription)-PCR

Total RNA was extracted from all cell lines using the TRIzol® RNA purification reagent. RNA quantity and purity were determined by using a NanoDrop ND-1000. Total RNA (1 μg) from each sample was reverse transcribed and real-time RT–PCR measurements were performed as described previously^47^ using an Mx3000 P apparatus (Agilent, CA, USA) with the corresponding SYBR Green kit. PCR primers were designed with Primer 3 (Agilent, CA, USA). Gene expression was normalized to the TATA box Binding Protein (TBP) and GAPDH (GlycerAldehyde-3-Phosphate DeHydrogenase). The sequence of primers used is indicated in Table S1.

### Statistics

All data are expressed as the mean ± SD. All data represent at least 3 independent experiments, and representative findings are shown. Students’ *t*-test was applied to estimate differences between the groups. *p < 0.05; **p < 0.01; ***p < 0.005.

## Electronic supplementary material

Supplementary Information
