# Supplementary material for: WISP1/CCN4 inhibits adipocyte differentiation through repression of PPARγ activity
Source: Sci Rep. 2017 May 11;7:1749. doi: 10.1038/s41598-017-01866-2 (PMC5431985; doi:10.1038/s41598-017-01866-2)
Supplement: Supplementary file 1 — Supplementary Information [file 41598_2017_1866_MOESM1_ESM.pdf]

Supplementary Information

WISP1/CCN4 inhibits adipocyte differentiation through repression of PPARγ activity

Nathalie Ferrand, Véronique Béreziat, Marthe Moldes, Maurice Zaoui, Annette K. Larsen and Michèle Sabbah

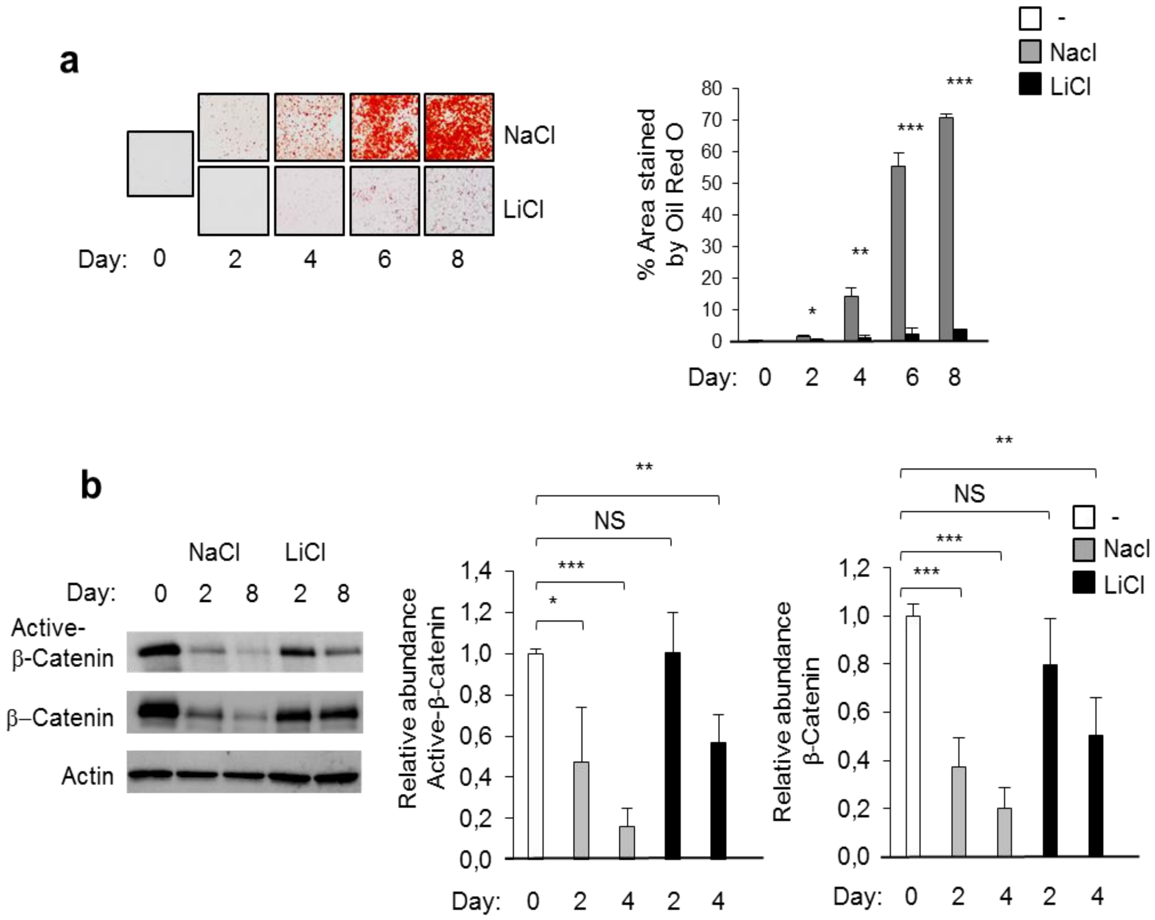

Fig. S1: Stimulation of Wnt signaling inhibits adipocyte differentiation.

3T3-F442A preadipocytes were exposed to MDI media supplemented with 25 mM Lithium Chloride (LiCl) or, as negative control, 25 mM Sodium Chloride (NaCl). (a) The differentiation process was monitored by microscopic imaging after Oil Red O-staining. Subsequently, Oil Red O-positive areas were measured by image analysis. (b) The expression of total or active β-catenin following Licl or NaCl treatment was assessed by immunoblotting. Immunoblot signals were quantified by densitometry, and normalized with actin. The values indicate the changes for the indicated sample compared to the vehicle. All values are representative of data from 3 independent experiments each performed in duplicate. Results are presented as means ± SEM * $p< 0.05$; ** $p<0.01$; *** $p < 0.005$.

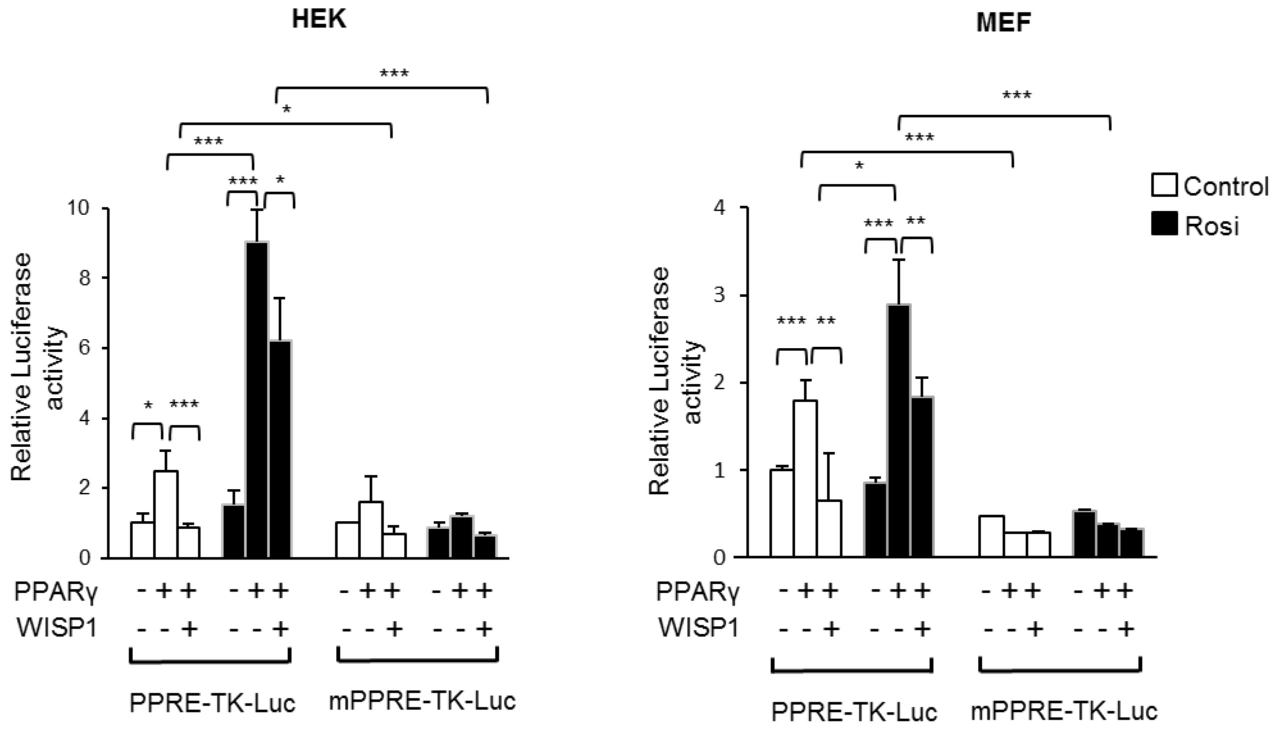

Fig. S2 : The Influence of WISP1 on PPARγ transcriptional activity is specific for PPARγ. HEK or MEF cells were transfected with 0.1 µg of wild-type (PPRE-TK-Luc) or mutated PPRE-TK-Luc (mPPRE-TK-Luc) alone or in combinations with 0.1 µg of PPARγ with or without 0.8 µg of WISP1 expression vectors along with RSV-β-galactosidase construct (0.02 µg) used as an internal control. Five hours after transfection, cells were treated (black bars) or not (open bars) with 1 µM rosiglitazone for 24 hours and assayed for luciferase and β-galactosidase activities. The results represent the average of at least three independent experiments each done in triplicate. Results are presented as means ± SEM. * $p< 0.05$; ** $p<0.01$; *** $p < 0.005$.

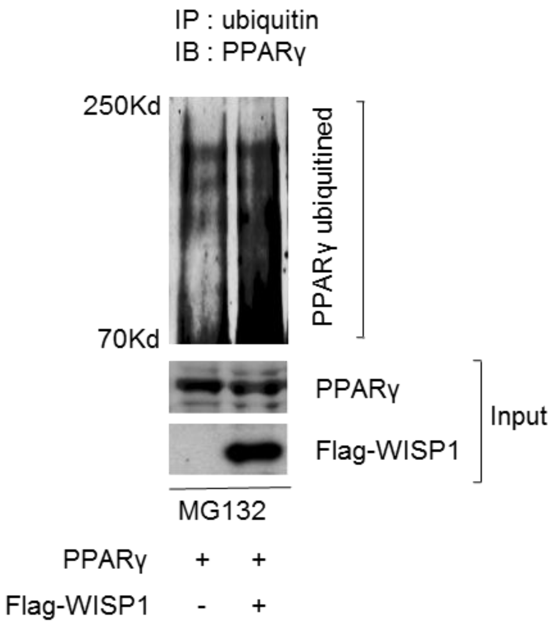

Fig. S3: WISP1 increases PPARγ ubiquitination. MEF cells transfected with the indicated expression vectors were treated with MG132 for 5 h before harvested and lysed, followed by immunoprecipitation using ubiquitin antibodies and Western blotting with polyclonal anti-PPARγ antibodies. Protein expression was determined by direct immunoblotting (Input).

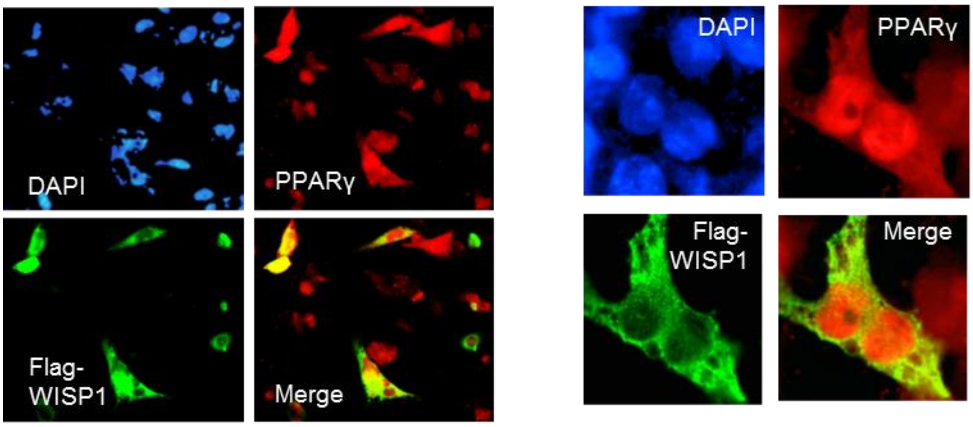

Fig. S4: Colocalization of PPARγ and WISP1. MEF cells were transfected with PPARγ and Flag-WISP1 expression vectors. The cells were immunostained with anti-PPARγ (red) and anti-Flag-WISP1 (green) antibodies and the nucleus was stained with DAPI. The nucleus, PPARγ and Flag-WISP1 were visualized by fluorescence microscopy.

Target Genes	Forward Sequence (5’-3’)	Reverse Sequence (5’-3’)
WISP1	CTGGACAGAAAAGGGCATGT	AGGAAGGAGGGGAAATCTCA
DLK1	AATAGACGTTCGGGCTTGCA	GGAGCATTCGTACTGGCCTTT
CEBPD	ACGACGGAGAGCGCCATC	TCGCCGTCGCCCCAGTC
PPARG	TTTTCAAGGGTGCCAGTTTC	AATCCTTGGCCCTCTGAGAT
ADIPOQ	GTTGCAAGCTCTCCTGTTCC	TCTCCAGGAG TGCCATCTCT
LPL	TTCAACCACAGCAGCAAGAC	CTGGATAATGTTGCTGGGC
FABP4	TTTCCTTCAAATGGGCGTG	CATTCCACCACCAGCTTGTC
CD36	GAATGGGCTGTGATCGGAAC	ACGTCATCTGGGTTTTGCAC
TBP	CCCTTGTACCCTTCACCAATGAC	TCACGGTAGATACAATATTTTGAAGCTG
GAPDH	AACTTTGGCATTGTGGAAGG	ACACATTGGGGGTAGGAACA

Table S1: Real-Time RT-PCR Primer Sequences
